# Funny waves in repolarisation and tachycardia in a patient suspected for Brugada syndrome

**DOI:** 10.1007/s12471-019-1292-8

**Published:** 2019-05-21

**Authors:** L. Jordaens, L. Timmers, P. Goethals

**Affiliations:** 1 Kliniek Sint Jan, Brussels, Belgium; 2 Universitair Ziekenhuis Gent, Ghent, Belgium

## Answer

**Fig. 1:**
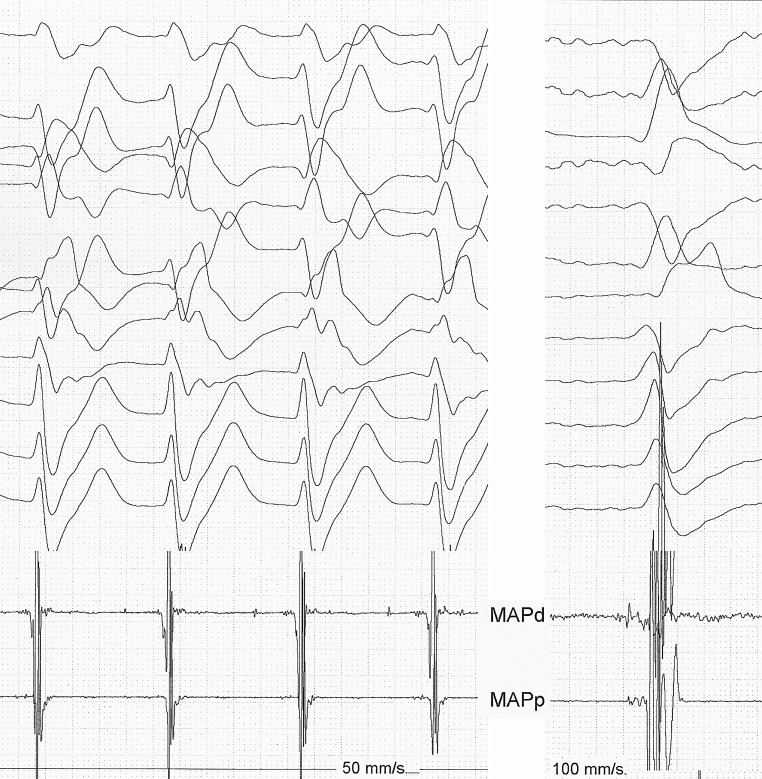
Recording (MAPd) on the site of ablation during tachycardia (*left*) and during sinus rhythm. A discrete potential precedes the QRS complex during tachycardia and is merged within the QRS complex during sinus rhythm

**Fig. 2:**
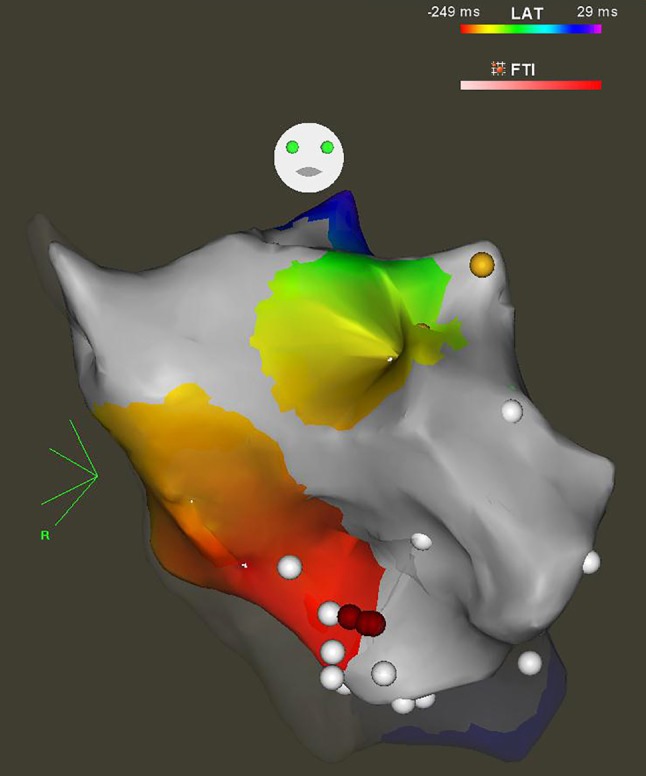
Electro-anatomical map of the left ventricle, showing the septal ablation site (*red dots*)—anterior view

The resting electrocardiogram (ECG) is diagnostic for Brugada syndrome [1]. His 2 brothers both had ECG patterns typical for Brugada syndrome, his mother had drug-induced Brugada syndrome. His father was from East Asian descent. The waves in V2 are indeed suggestive of an epsilon wave, which might be a sign for arrhythmogenic right ventricular dysplasia, and hence an overlap situation [2]. However, there were no clinical, nor other signs for this disease, and the cardiac magnetic resonance imaging (MRI) was entirely normal. Depolarising abnormalities are observed in 13% of patients with spontaneous or drug-induced Brugada syndrome, without any evidence of cardiomyopathy [2]. The tachycardia was a left-sided posterior fascicular tachycardia and was easily ablated on a site with a Purkinje potential (Fig. 1 and 2). A dual-chamber implantable cardioverter-defibrillator was implanted, given the bradycardia and conduction disease. With a follow-up of 3 years, no events have been recorded. Fascicular tachycardia has in principle no relation with Brugada syndrome, which mainly affects the right ventricular outflow tract [3]. That some molecular common link may exist cannot be excluded. Genetic analysis showed 2 missense variants in the SCN5A gene, and one TMEM43 variant, all with unclear relation to his disease, given his East Asian roots.
